# The Work of London and Provincial Hospitals

**Published:** 1914-10-03

**Authors:** 


### HOSPITALS AND THE WAR.

(From our Special Correspondents.)

### The Work of London and Provincial Hospitals.

### CARDIFF AND DISTRICT: SECOND BATCH OF WOUNDED ARRIVE.

The second batch of wounded soldiers, numbering 119 and belonging to a variety of regiments, arrived at Cardiff last Sunday night for treatment at the Albany Road and Splott Road sections of the 3rd Western General Hospital. They had been conveyed first to the St. Nazaire Collecting Hospital, thence by the steamship *Carisbrook Castle* to Southampton; thirty-four were medical cases, the remainder surgical. As usual, the great majority of the latter were shrapnel wounds in the lower limbs, and as testimony to the German shrapnel fire one soldier remarked that he was lying in a trench by the River Aisne for two whole days before he could be removed. The total received at Cardiff from the Front is now 239, of whom forty-nine have been discharged "cured."

A magnificent ovation was accorded the men by the great crowd which assembled along the whole of the route. Private cars and covered motor-vans fitted up as ambulance waggons were in readiness to receive the wounded as they were transferred on ambulances under the able direction of Major Ewen Maclean, M.D. The Voluntary Aid Detachments rendered splendid service. On the platform were the Lord Mayor, Dr. J. Robinson, and other well-known workers and officials. The men showed great cheerfulness, and they carried many trophies. On arrival at the sections they were received by Colonel Hepburn, V.D., the officer commanding this division.

### CHATHAM DISTRICT: ANGLO-GERMAN HOSPITAL TRAIN.

A hospital train from Harwich recently arrived at Chatham with forty-eight wounded British and German sailors. The Germans were survivors of the cruiser *Mainz* and the *Koenigin Luise*, and men who took part in the Heligoland battle. The men of the *Mainz*, five in number, who were able to walk to the station, were placed together in one compartment with an escort of the Bedfordshire Regiment, under the command of Lieutenant Lord Cranborne, eldest son of the Marquis of Salisbury. The British sailors all belonged to the three cruisers sunk last week. Upwards of a hundred wounded men belonging to various regiments arrived at Chatham by special train from Southampton on September 21, and were conveyed to the Drill Hall, Fort Pitt Road, which has been converted into a hospital. A few German prisoners were also brought in by the same train and taken to Fort Pitt under an armed guard. A number of wounded soldiers have been transferred from Fort Pitt Hospital to the care of the Strood Voluntary Aid Detachment. The temporary hospital is on the Frindsbury Road. The Earl of Darnley has been appointed to the command of the Kent Voluntary Hospitals, Territorial Force.

### LEICESTER: THE PROGRESS OF THE WOUNDED.

Rapid progress has been made by the builders with the remaining buildings required to bring the 5th Northern General Hospital up to the R.A.M.C. standard of 520 beds, with the result that the commanding officer now reports 460 beds available. With the arrival of the second trainload of wounded soldiers, the number of beds occupied was brought up to about 230. Several of the new patients are suffering from wounds of a dangerous nature, and there are two very severe cases of tetanus; but the majority are making good progress, and all express themselves as highly gratified with the care and attention which are being shown them. Additional nurses were mobilised to meet the increased number of patients. Another member of the R.A.M.C. (T.) staff of the hospital has been called up in the person of Captain F. Bolton Carter, one of the surgeons to the Royal Infirmary.

The entertainments arranged by the chaplain (Rev. W. C. Luxmoore) on Tuesday and Saturday evenings are much appreciated by the patients and staff. Great interest was taken by the hospital's little self-contained colony last Saturday in a football match between a hospital team and a team from the chaplain's parish. A number of Belgian refugees—mostly women and children—have been provided with homes in the town and county.

### PERTH: NEW INFIRMARY.

The directors of this institution, in addition to receiving for treatment serious cases of accident or illness occurring among the troops mobilised in the district, have agreed to set apart forty-two beds for the treatment of wounded soldiers sent home from the seat of war. To this end convalescent cases are being discharged from the institution, and only cases requiring urgent treatment admitted in the meantime so that the number of beds offered may be available on the shortest notice. This offer on the part of the infirmary directors is of special importance in view of the fact that they have agreed to treat cases of wounded sent to them free of charge.

### SHEFFIELD: SECOND CONVOY ARRIVE.

The second convoy of wounded to be dealt with at the 3rd Northern General Hospital arrived at Sheffield in the early hours of Friday, September 25. They number exactly the same as the first convoy, 125, and are representative of English, Scottish, Irish, and Welsh regiments that have been taking part in the big battle of Aisne. Of the 125 there are 120 wounded and five sick cases. The type of case is indicative of the severity of the fighting operations through which the men have passed far more than the cases of the first convoy. Shrapnel wounds predominate, and in several cases surgical treatment has been necessary. Satisfactory progress is reported, though, as is inevitable from the conditions prevailing on the battlefield, to have to deal with so many suppurating wounds, received in most cases four or five days previously, naturally presents difficulties and contrasts with the cases of average civilian practice. Notwithstanding the long and tedious journey by motor-waggon, ammunition waggon, and other transports over bad roads, slow railway journeys in often ill-adapted carriages or even trucks, followed by the cross-Channel trip from Havre to Southampton, and then a further five or six hours' railway journey, the men, on arriving at Sheffield, showed very few ill-effects, and a member of the hospital staff expressed the opinion that having regard to all the difficulties of transport it was managed wonderfully well. When they reached Sheffield at two o'clock in the morning a small crowd even at that hour was awaiting them. They were expeditiously placed in the five motor-ambulances and six Corporation motor-'buses by the R.A.M.C. men, and were soon in the wards of the Education Committee's Training College, now converted into a hospital. Of the 125 cases in the first convoy that came to Sheffield a month ago only about a dozen now remain, the majority having rejoined their regiments.

### TORQUAY: A "PALATIAL" HOSPITAL.

Mr. Paris Singer's residence at Oldway, Paignton, has been transformed into a wonderfully fitted-up hospital. The arrangements were carried out by the American Women's War Committee. It contains 150 beds, an up-to-date operating-theatre, *x*-ray and anæsthetic rooms. Every requirement possible has been provided; in fact, it is more like a palace than a hospital. "I am sure," writes our special correspondent, "it surpasses any other in the West of England." The principal medical officer is J. E. Lane, F.R.C.S.; consulting physician, Sir William Osler, M.D. The physicians and surgeons are Sir Randolph Smith, Messrs. A. E. Carver, W. W. Stabb, L. Bennett, W. M. Steele, H. C. Adams. The resident medical officer is Rupert Farrant, F.R.C.S.; matron, Miss Gertrude Fletcher, and a large staff of trained nurses.

Lieut.-Col. R. C. Gunning, of Redhill, Paignton, has been appointed by the War Office as commandant of the hospital. On Sunday evening 130 wounded British soldiers (part of the 800 landed at Southampton in the morning) reached Paignton.

The patients were removed to "Oldway" in Red Cross ambulances and motor-vans. They were met at the station by Mr. Singer and Dr. J. E. Lane.

### TUNBRIDGE WELLS: EMERGENCY WORK AT MIDNIGHT.

The accommodation provided by the General Hospital at Tunbridge Wells was called up by the military authorities for the reception of wounded last Tuesday. The hospital's authorities were informed that some twenty patients were coming from Chatham in motor-cars, kindly lent by various people in this neighbourhood. The St. John Ambulance men, under Chief Officer E. R. Hickmott, undertook the transport of the cases from Chatham to Tunbridge Wells. The General Hospital is working in cordial co-operation with the medical officers in charge of the Territorials encamped at Tunbridge Wells and also at Crowborough. An urgent case was admitted from the latter camp on Thursday last week at 11 p.m. and immediately operated upon. The case was brought from the camp in the Bishop of London's motor-car, his lordship being on duty there.

### WOUNDED FROM THE AISNE AT THE WEST HAM HOSPITAL.

The West Ham and Eastern General Hospital has been affiliated with the Royal Herbert Hospital at Woolwich for the purpose of the reception and the treatment of wounded soldiers from the war. Last week the chairman, Mr. T. A. Cook, received an intimation from Colonel R. R. Simpson, A.D.M.S., Woolwich district, to be prepared to receive twenty wounded cases. Having received many offers of help in such a case, the executive of the West Ham Hospital offered to convey the patients to the hospital, and from the many offers selected six light kindly lent motor-vans.

Punctually at two o'clock these vehicles assembled in the courtyard of the Royal Herbert Hospital. There were twelve "cot" cases—*i.e.* those who were unable to sit up, and had to be conveyed on stretchers and mattresses to the hospital—and eight cases able to walk and sit. The "cot" cases were carefully carried and laid on new mattresses provided by the West Ham Hospital in the various light motor-vans, whose drivers had instructions to drive carefully over a previously selected route, where attention was given to the condition of the roads as well as to the nearness of approach and freedom from traffic. The chairman of the hospital was the first to leave with two of the less severely wounded, a sergeant, and an orderly, and thus arrived at the hospital as early as any other conveyance. Here the porters were waiting, and the police had a clear road ready for the reception of the various cars and vans as they came up. The Marlborough Ward had been cleared and cleaned and was in perfect readiness for the reception of the whole twenty cases. As each car or van arrived the cases were speedily conveyed to the lift and thence to the ward, where an ample staff of nurses and sisters were waiting to receive them, as well as three honorary surgeons, the house-surgeon, and some of the honorary physicians. Within an hour of leaving the Royal Herbert Hospital all the "cot" cases were carefully tucked away in beds, and the other cases were sitting around a fire having a splendid tea. They much enjoyed the bread and butter and fresh-made tea provided. On Sunday the Rev. J. Merrin, honorary chaplain to the hospital, conducted a short service in the ward, which was much appreciated. The thanks of the committee are due to all who assisted in so easily conveying these gallant men.

Most of the men came, as far as can be ascertained, from [illegible]e battle of the Aisne, and many thrilling stories are told of the fighting.

